# Mortality of type 2 diabetes in Germany: additional insights from Gompertz models

**DOI:** 10.1007/s00592-024-02237-w

**Published:** 2024-03-11

**Authors:** Oliver Kuss, Jens Baumert, Christian Schmidt, Thaddäus Tönnies

**Affiliations:** 1 Institute for Biometrics and Epidemiology, German Diabetes Center, Leibniz Institute for Diabetes Research at Heinrich Heine University Düsseldorf, Düsseldorf, Germany; 2 Centre for Health and Society, Faculty of Medicine, Heinrich Heine University Düsseldorf, Düsseldorf, Germany; 3 German Center for Diabetes Research, Partner Düsseldorf, Munich-Neuherberg, Germany; 4 Department of Epidemiology and Health Monitoring, Robert Koch Institute, Berlin, Germany

**Keywords:** Epidemiology, Type 2 diabetes, Mortality, Germany, Gompertz distribution

## Abstract

**Aims:**

The Gompertz law of mortality proclaims that human mortality rates in middle to old ages grow log-linearly with age and this law has been confirmed at multiple instances. We investigated if diabetes mortality in Germany also obeys to the Gompertz law and how this information helps to communicate diabetes mortality more intuitively.

**Methods:**

We analyzed all statutory health-insured persons in Germany in 2013 that were aged 30 years or older. Deaths in 2014 were recorded and given in 5-year age groups. We fitted weighted linear regression models (separately for females and males and for people with and without diabetes) and additionally computed the probability that a person with diabetes dies before a person of the same age and sex without diabetes, and the “diabetes age”, that is, the additional years of mortality risk added to an individual’s chronological age due to diabetes-related excess mortality.

**Results:**

We included *N* = 47,365,120 individuals, 6,541,181 of them with diabetes. In 2014, 763,228 deaths were recorded, among them 288,515 with diabetes. Diabetes mortality followed nearly perfectly Gompertz distributions. The probability that a person with diabetes dies before a person without diabetes was 61.9% for females and 63.3% for males.

**Conclusions:**

Diabetes mortality for females and males aged 30 years or older in Germany in 2014 followed the Gompertz law of mortality. The survival information of the population with diabetes during a large part of the lifespan can thus be reduced to the two parameters of the Gompertz distribution.

## Introduction

In 1825, the British actuary Benjamin Gompertz discovered a strikingly simple relation between the mortality rate and age [1]. He had analyzed death records in Sweden, England and France and found that the natural logarithm of the mortality rate between ages of 20 and 60 years grows linearly with age [2].

In mathematical terms, the “Gompertz Law of Mortality” can be written as

$$\upmu \left( x \right) \, = \alpha e^{\beta x} ,$$

where µ(*x*) denotes the mortality rate, that is, the hazard of death at age *x*, and *α* and *β* are constants that can be estimated from reported life tables or other survival information. That is, the Gompertz law effectively reduces the survival information of a complete population during a large part of the possible lifespan to just two parameters (*α* and *β*). In addition, it was found that the Gompertz law applies nearly universally, especially also across time, and even across species [3], but of course with varying age ranges.With respect to mortality among people with diabetes there is some evidence for the log-linear association of mortality rates with age [4–8] although this has not always explicitly attributed as following the Gompertz law.

In the following we report on an analysis of a claims data set from all statutory health-insured persons in Germany in 2013 aged 30 years or older, with the vital status in the study population ascertained from the same data set in 2014. This work has several aims. First, we check if diabetes mortality in Germany also follows the Gompertz law. Second, as we find this to be the case, we propose two underused measures for describing diabetes mortality, (i) the probability that a person with diabetes dies before one without diabetes and (ii) the “diabetes age”, that is, the additional years of mortality risk added to an individual’s chronological age if diabetes is present. Third, we quantify the strength of the association between mortality rates and age and thus the goodness of the Gompertz fit by computing R^2^ statistics. Finally, we assess if two other mechanistically plausible distributions (Weibull and Logistic) are superior to the Gompertz fit.

## Methods

We use the data set from a recent publication on excess mortality in adults with documented diabetes in Germany [9]. This includes all statutory health-insured persons in Germany in 2013 (roughly 90% of the German population) that were aged 30 years or older. Diabetes was defined by the ICD-10 codes E10-E14, documented in 2013 in at least two-quarters on an outpatient setting or at least once on an inpatient setting.

The vital status of the study population was assessed in 2014, and given in 5-year age groups (30– < 35, 35– < 40,…,> 95). We applied two modifications to the original data set. First, we modified the denominator of the mortality rates within each group of age, sex and diabetes state by adding only half of the observation time (0.5 years) under risk for all deaths to the denominator. This reflects the assumption that death times are uniformly distributed across the observation time. Second, instead of using 5-year age group as a categorical variable in the model fit, we used the mean age of death in the respective group as a continuous variable. We additionally accounted for the fact that within age groups people at older ages are more likely to die than people at younger ages. To this task, we used the most recent (2017) additive correction factors for Germany as proposed by the Human mortality database [10], where we applied identical correction factors for people with and without diabetes. The full data set is given in Table 1, where the corrected mean ages of death are given in the second column. For example, in the age group 70–74 for females, we used the mean age of death of 72.64 years, instead of 72.5 years which would have been the midpoint of the interval.

**Table 1:** Full analysis data set for the four groups of diabetes state and sex

Age group	Mean age at death	Number of deaths in 2014	Number of survivors in 2014	Mortality rate per 1000 persons
No diabetes, Female				
30–34	32.66	612	2,041,495	0.30
35–39	37.72	881	1,939,888	0.45
40–44	42.70	1628	2,107,408	0.77
45–49	47.70	3555	2,754,706	1.29
50–54	52.74	6232	2,787,650	2.23
55–59	57.67	7902	2,312,260	3.41
60–64	62.64	10,226	1,950,936	5.23
65–69	67.67	11,404	1,451,002	7.83
70–74	72.64	18,929	1,608,154	11.70
75–79	77.69	28,674	1,468,599	19.34
80–84	82.69	36,859	884,115	40.84
85–89	87.56	54,580	602,540	86.66
90–94	92.31	52,373	280,705	170.7
> 95	96.95	20,553	55,476	312.6
No diabetes, Male				
30–34	32.64	1198	1,932,965	0.62
35–39	37.65	1547	1,768,244	0.87
40–44	42.71	2622	1,872,286	1.40
45–49	47.68	5717	2,436,612	2.34
50–54	52.74	10,115	2,439,861	4.14
55–59	57.69	13,255	1,957,523	6.75
60–64	62.66	16,813	1,553,716	10.76
65–69	67.62	17,309	1,103,558	15.56
70–74	72.61	26,659	1,165,921	22.61
75–79	77.61	34,891	1,002,363	34.21
80–84	82.60	34,466	520,173	64.13
85–89	87.45	33,130	264,202	118.0
90–94	92.21	18,074	76,532	211.2
> 95	96.86	4509	10,336	358.1
Diabetes, Female				
30–34	32.66	45	22,168	2.03
35–39	37.72	59	31,679	1.86
40–44	42.70	195	51,784	3.76
45–49	47.70	446	96,031	4.63
50–54	52.74	1111	158,758	6.97
55–59	57.67	2132	229,959	9.23
60–64	62.64	3703	327,596	11.24
65–69	67.67	5518	352,288	15.54
70–74	72.64	11,063	479,304	22.82
75–79	77.69	21,773	597,348	35.80
80–84	82.69	28,747	427,773	65.02
85–89	87.56	35,931	279,490	120.8
90–94	92.31	28,962	120,278	214.9
> 95	96.95	8806	20,149	358.7
Diabetes, Male				
30–34	32.64	78	18,251	4.26
35–39	37.65	133	30,218	4.39
40–44	42.71	328	61,490	5.32
45–49	47.68	1015	131,778	7.67
50–54	52.74	2337	227,310	10.23
55–59	57.69	4582	310,250	14.66
60–64	62.66	8056	398,011	20.04
65–69	67.62	10,769	406,064	26.17
70–74	72.61	19,909	500,685	38.99
75–79	77.61	30,090	513,675	56.91
80–84	82.60	28,810	291,350	94.23
85–89	87.45	22,370	132,780	155.4
90–94	92.21	9660	32,485	258.9
> 95	96.86	1887	3714	405.2

For primary statistical analysis we fitted Gompertz models in four groups, that is, separately for females and males, and for people with and without diabetes. Following the proposal of Tai/Noymer [11], we used weighted linear regressions with the natural logarithm of the mortality rate in the respective age group (Log[MR]) as the outcome, the mean age of death within age groups as the only (continuous) covariate, and the absolute number of deaths D within age groups as weights. To be concrete, the model is a linear regression model

$${\text{Log}}\left[ {{\text{MR}}} \right]\left( X \right) \, = {\text{ Log}}(\alpha ) + \beta {\text{x }} + \, \varepsilon ,$$

with *ε* the residual error. The regression parameter *β* for the mean age of death then equals the Gompertz parameter *β*. The Gompertz parameter *α* is computed via exponentiation from the model's intercept Log(*α*). The four weighted linear regression models were fitted by maximum likelihood with a common Log-likelihood function to enable comparison across groups while properly accounting for the full estimation uncertainty.

To assess the fit of the Gompertz models to the observed Log(MR)s, we drew scatterplots of Log(MR) versus age. In the case of mortality being actually Gompertzian, Log(MR) and mean age would be linearly related and we computed weighted (using the absolute number of deaths as the respective weight) *R*^2^ statistics to assess this linearity and the variation on Log(MR) that is explained by the regression on age. In a second effort to assess the Gompertz fit, we computed weighted linear regression models assuming the distribution for age of death to follow a Weibull or a logistic distribution. Both distributions have been described as logically plausible to model human mortality at middle and higher ages [12, 13]. In the Weibull case, the weighted linear regression model has to use the natural logarithm of the mean age of death as the single covariate. In the logistic case, the outcome of the weighted linear regression model is not the Log[MR], but the Logit[MR], that is, Log[MR/(1-MR)].

In addition, we also report on two (at least in our view) underused measures to communicate diabetes mortality, first, the probability that a person with diabetes (and the same age and sex) dies before a person without diabetes and, second, the “diabetes age”.

The probability that a person with diabetes dies before a person without diabetes ranges between 0 and 100%. A value of 50% (or 0.5) indicates that a person with diabetes would die before a person without diabetes in half of the cases. This would correspond to the toss of a coin and to no association between diabetes and mortality. For Gompertz distributions this probability can only be computed by numerical integration and Saracoglu/Kaya [14] give the respective formula.

The “diabetes age” is defined as the additional years of mortality risk added to a person’s chronological age if diabetes is present. It is thus only defined for persons with diabetes and it can be interpreted for example as follows: “Your diabetes age corresponds to the age of a person without diabetes that has the same mortality risk. So if your chronological age is 60 years, but your diabetes age is 67 years, this means that you are in the same risk category as a 67-year old without diabetes.” [15]

Calculating diabetes age is straightforward by using elementary formulas for the predicted Gompertz log hazards for people with and without diabetes at each respective chronological age.

**Fig. 1:**
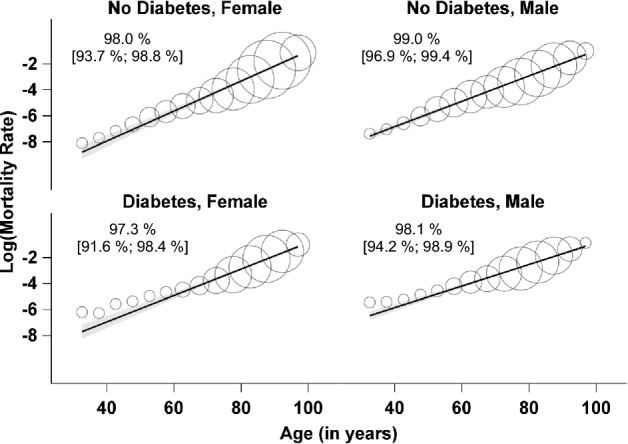
Scatterplots of Log(MR) versus age in the four groups of diabetes state and sex. Regression lines are given with 95% confidence intervals and originate from the respective weighted regression model using the absolute number of deaths in the respective age group as weights. Bubble sizes are proportional to the respective weights. Insets give weighted *R*^2^ statistics with their 95% confidence intervals

All analyzes were performed in SAS (SAS Institute Inc., Cary, NC, USA), Version 9.4, where we used the NLMIXED procedure for the model fit (Fig. 1).

This study used only published, aggregated data and so we did not seek for the vote of a ethical committee.

## Results

The data set comprised 47,365,120 individuals, 6,541,181 of them with diabetes. In 2014, 763,228 deaths were observed, among them 288,515 with diabetes. In Table 2, we give the results from fitting the Gompertz model in the four groups of sex and diabetes state. Figure 1 shows the scatterplots of Log(MR) versus age together with regression lines from the respective weighted fit. The association between Log(MR) and age is remarkably linear and the weighted R^2^ statistics are always larger than 97%, thus almost perfectly pointing to Gompertz distributions for age of death in all four groups. As can be seen from weighted R^2^ statistics in Table 3, the Weibull and the logistic distribution also give excellent fits, however, they do not improve the Gompertz fit, with the respective weighted R^2^ statistics always being smaller.

**Table 2:** Results from fitting Gompertz models in the four groups of diabetes state and sex. Confidence intervals are only given if they change before the third decimal place

	Female		Male	
	No diabetes	Diabetes	No diabetes	Diabetes
Gompertz *α*	0.0000035	0.000016	0.000021	0.000102
Gompertz *β*	0.115	0.102	0.098	0.083
Weighted R^2^ statistics for the regression of Log(MR) on mean age of death	98.0% [93.7%; 98.8%]	97.3% [91.6%; 98.4%]	99.0% [96.9%; 99.4%]	98.1% [94.2%; 98.9%]
Probability that a person with diabetes dies before a person without diabetes	61.9%	61.9%	63.3%	63.3%
Gompertz *α* (Poisson Fit)	0.0000031	0.000013	0.000021	0.000098
Gompertz *β* (Poisson Fit)	0.117	0.104	0.097	0.083

**Table 3:** Results from fitting Weibull and logistic models in the four groups of diabetes state and sex

	Female		Male	
	No diabetes	Diabetes	No diabetes	Diabetes
Weibull fit:				
Weighted R^2^ statistics for the regression of Log(MR) on Log(mean age of death)	93.6% [80.6%; 96.2%]	92.9% [78.7%; 95.8%]	96.0% [87.5%; 97.6%]	94.3% [82.6%; 96.6%]
Logistic fit:				
Weighted R^2^ statistics for the regression of Logit(MR) on mean age of death	97.1% [91.0%; 98.3%]	96.0% [87.8%; 97.6%]	98.3% [94.7%; 99.0%]	96.9% [90.2%; 98.1%]

**Fig. 2:**
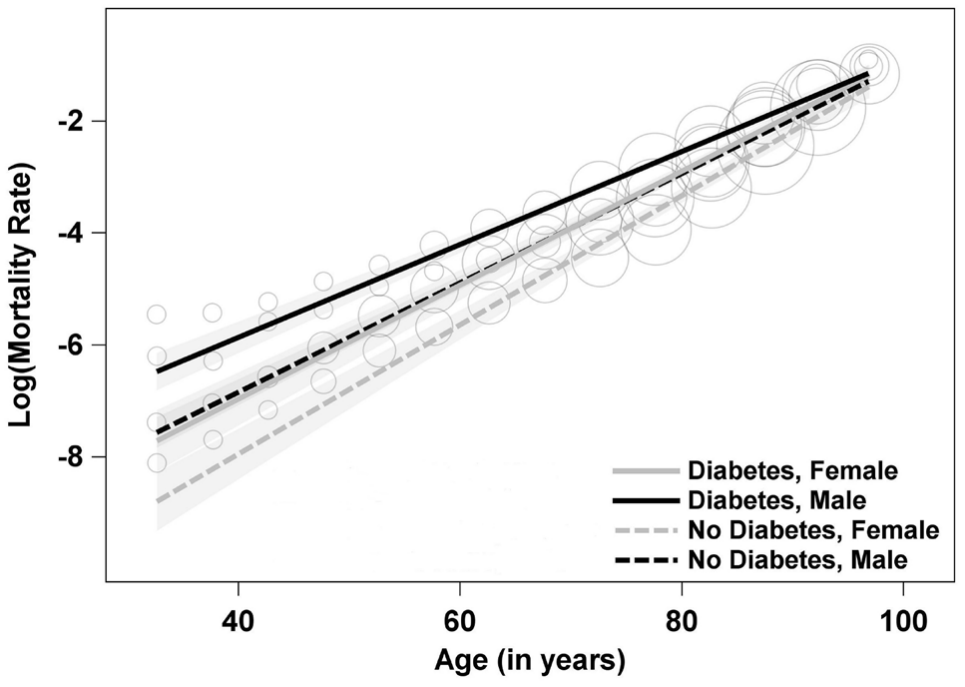
Overlaid scatterplots of Log(MR) versus age in the four groups of diabetes state and sex. Regression lines are given with 95% confidence intervals and originate from the respective weighted regression model using the absolute number of deaths in the respective age group as weights. Bubble sizes are proportional to the respective weights

**Fig. 3:**
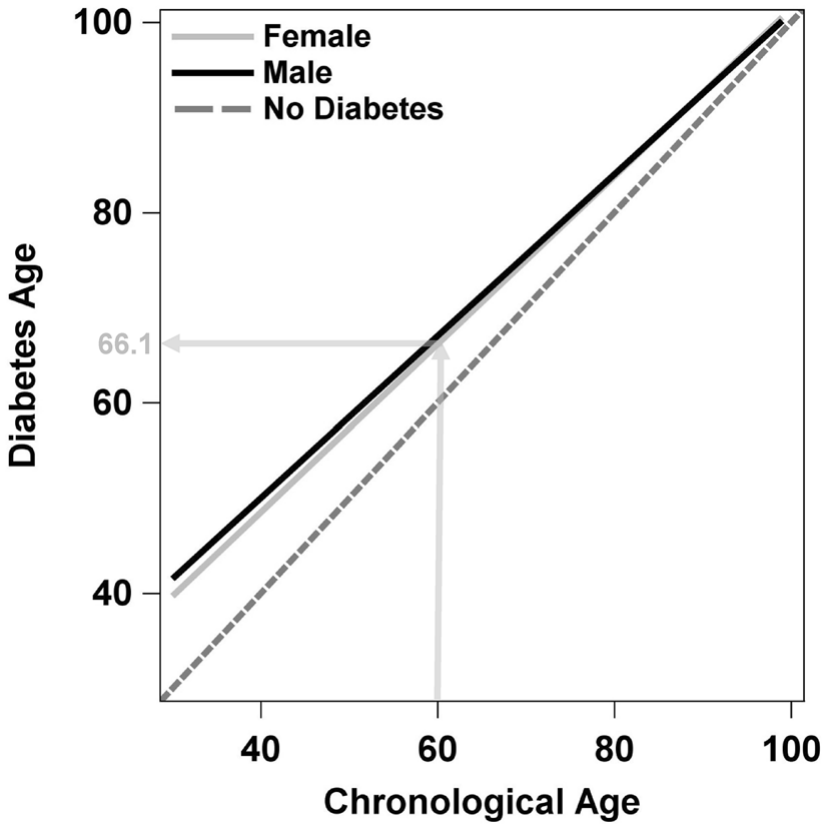
Diabetes age for females and males. The light gray lines give the example of a female with diabetes and a chronological age of 60 years. Her diabetes age is 66.1 years

Figure 2 shows the four scatterplots overlaid, and we see that mortality of females *with* diabetes is nearly identical to that of males *without* diabetes. The probability that a female/male with diabetes dies before a female/male without diabetes (and the same age) is 61.9/63.3% (Table 2). In Fig. 3 we report on the diabetes age. This declines with chronological age and is, for example, 66.1/67.0 years for a female/male with diabetes and a chronological age of 60 years.

## Discussion

Diabetes (as well as non-diabetes) mortality rates in Germany for both sexes follow nearly perfectly Gompertz distributions. That is, the full information on diabetes (and non-diabetes) mortality in Germany can be summarized in just two numbers and a variety of easy accessible numbers and graphical displays can be derived thereof. We gave two of them, first, the probability that a person with diabetes dies before one without diabetes and, second, the “diabetes age”, that is, the additional years of mortality risk added to an individual’s chronological age if diabetes is present. Besides this parsimony (i.e., reducing the survival information of a population to just two parameters), Congdon [16] lists a number of additional advantages when using parametric distributions for reporting mortality figures:

1. *Smoothness:* Random fluctuations due to small sample sizes or fluctuations in age regions with low numbers of observed deaths (e.g., in very old ages) are smoothed.
2. *Interpolation:* Mortality rates for any specific age can be analytically derived which is especially useful when only coarse information (e.g.,, in 5-year or even 10-year age groups) is available.
3. *Comparison:* Comparison between different populations, observation times, or modes of data collection is facilitated.
4. *Trends and forecasting:* The assessment of trends over time and forecasting into the future is facilitated.

In addition, using parametric distributions also enhances biological plausibility of age-at-death distributions. The regularly used models which use Poisson assumptions for the number of deaths in age groups necessarily assume that the hazard within each age group is constant, and only jumps to another (then again constant) level when proceeding to the next age group. Of course, this assumption of mortality as a step function of age is biologically highly implausible and we instead expect mortality to develop smoothly and without jumps in the age course. We yet assume that differences between parametric fits and Poisson piecewise constant fits would be minor, at least with age intervals being not too large. Indeed, Tai/Noymer [11] also proposed a Poisson model in their comparison of models for Gompertz mortality and we fitted this model to our data set. Results for the Gompertz parameters *α* and *β* are given in Table 2 and, as expected, deviate only marginally from the results of the weighted linear fit.

In terms of the actual analysis, the primary strength is that it uses a large data set, covering about 90% of the German population. In addition, mortality rates as seen in our data showed good agreement with data from official death statistics [9].

With respect to diabetes age, it is important to note that this number conveys different information as a reduction in life expectancy (RLE) although both measures use the same scale of absolute time. Imagine two persons who are identical in every way, except that one has diabetes, the other not. When referring to the RLE, then for the person having diabetes, this is expected to take, say, X years off the length of their life. When referring to the diabetes age, then for the person having diabetes, this gives them the annual chance of death of someone who is X years older [17].

Pang/Henley [18] gave some more insights into the relation between diabetes age and RLE and showed that the diabetes age is an upper bound for the RLE and thus always larger, but that this is only true for proportional hazards. Proportional hazards would mean in our case that the lines in Fig. 2 for people with and without diabetes within sexes would be parallel. This is obviously not that case and we rather see the well-known compression of mortality (converging log-linear hazards) in the diabetic as well as in the non-diabetic population. As such, it is difficult, if not impossible, to derive any relations between the diabetes age and the RLE here which is of course another argument for using the diabetes age per se, and in addition to the reduction in life expectancy. However, and as shown by Heard et al. [17], there are also challenges in the interpretation of diabetes age when compared to the RLE or a less-distant-in-time measure like the hours of life expectancy lost each day. Following Heard et al. [17] it is advantageous to communicate diabetes age not in a change-in-age format (“Having diabetes makes you *X* years older”), but in a personal format (“Having diabetes and being *Y* years old, increases your diabetes age to *Y* + *X* years”). It might be a fruitful area of further research how the concept of diabetes age is understood in our target population of middle-aged to older people with diabetes.

To check if the relation between diabetes age and RLE is given despite the violation of the proportional hazard assumption we exemplarily looked for the RLE for a woman with diabetes and a chronological age of 60. As seen in Fig. 3, such a woman has a diabetes age of 66.1. In a previous analysis of our group [19] which used the same data set, the parallel RLE would be 3.7 years. In two external cohorts with similar observation times (around 2013) a RLE of 3.9 years was reported from Scotland [20], and of around 3 years from Sweden [21] for a 60-year old woman with diabetes. That is, despite the proportional hazard assumption not being fulfilled, the theorem of Pang/Henley might be at least approximately true and the diabetes age is larger than the RLE.

It is fair to point to some limitations of our analysis. Of course, our data report only on a single year (2013), and results might be different in previous or subsequent years. We could not distinguish the type of diabetes because the data contained implausible double diagnoses of type 1 and type 2 diabetes in the same person. The study data are limited to documented diagnoses, that is, no information about mortality of undiagnosed diabetes is available. Those who died with newly documented diabetes in 2014 are not detectable in the data set as cases and hence were erroneously counted as persons without diabetes. Finally, duration of diabetes (and other covariates) were not available and not accounted for.

In terms of the probability that a person with diabetes dies before a person without diabetes it is somewhat unsatisfactory that this cannot be given age-dependently. Instead, this probability is assumed constant and we have to interpret it as an averaged value across the full age range. To tackle this problem we fitted Gompertz models with decreasing age ranges by increasing the entry age. That is, we started with the full model (age range 30– > 95 years), then repeated the analysis for a smaller data set starting at the age of 35 years etc. This worked well and the estimated probabilities were rather stable and always near the values for the full age range for both sexes.

For future work it would be interesting to investigate if our results are generalizable to other situations, e.g.,, countries, observation times, modes of data collection, or in the time course.

In conclusion, diabetes mortality for females and males aged 30 years or older in Germany in 2014 follows the Gompertz law of mortality. The survival information of the population with diabetes during a large part of the lifespan can thus be reduced to the two parameters of the Gompertz distribution. In addition, the Gompertz distribution gives better fits than two competing, mechanistically also plausible distributions for the age at death. From the Gompertz distribution, further insightful figures can be computed and communicated. The probability that a female/male with diabetes dies before a female/male without diabetes (and the same age) is 61.9%/63.3%. Diabetes age declines with chronological age and is, for example, 66.1/67.0 years for a female/male with diabetes and a chronological age of 60 years.
